# Epidemiological characteristics of central nervous system tumors in children: a 5-year review of 3180 cases from Beijing Tiantan Hospital

**DOI:** 10.1186/s41016-022-00279-z

**Published:** 2022-05-12

**Authors:** Zhi-ming Liu, Chih-yi Liao, Heng Zhang, Zhe Han, Jun-mei Wang, Zhen-yu Ma, Chun-de Li, Jian Gong, Wei Liu, Tao Sun, Yong-ji Tian

**Affiliations:** 1 Department of Neurosurgery, Beijing Tiantan Hospital, Capital Medical University, Beijing, China; 2 China National Clinical Research Center for Neurological Diseases, Center of Brain Tumor, Beijing Institute for Brain Disorders, Beijing Key Laboratory of Brain Tumor, Beijing, China; 3 Department of Neuropathology, Beijing Neurosurgical Institute, Capital Medical University, Beijing, China

**Keywords:** Epidemiology, Central nervous system tumors, Pediatric, World Health Organization classification

## Abstract

**Background:**

To describe the epidemiological characteristics of central nervous system (CNS) tumors in children, based on the neurosurgery department of Beijing Tiantan Hospital.

**Methods:**

From January 2015 to December 2019, 3180 children were histopathologically diagnosed with CNS tumors based on the 2016 World Health Organization (WHO) classification of tumors. Patients were 0 to 15 years old. We analyzed age-related gender preferences, tumor locations, and the histological grades of the tumors. In addition, the epidemiological characteristics of the five most common intracranial tumors were compared to the previous studies.

**Results:**

In this study, intracranial and spinal tumors account for 96.4% (3066) and 3.6% (114) of all tumors, with a preponderance of supratentorial tumors (57.9%). Among all pediatric patients, low-grade tumors comprise 67.1% (2 135). The integral gender ratio of males to females is 1.47: 1 and the average age of patients is 7.59 years old. The five most common intracranial tumors are craniopharyngioma (15.4%), medulloblastoma (14.3%), pilocytic astrocytoma (11.8%), diffuse astrocytoma (9.8%), and anaplastic ependymoma (4.8%).

**Conclusions:**

Due to the lack of national data on childhood brain tumors, we used a large nationally representative population sample based on the largest pediatric neurosurgery center in China. We analyzed the data of the past 5 years, reflecting the incidence of CNS tumors in Chinese children to a certain extent, and laying a data foundation for subsequent clinical studies.

**Supplementary Information:**

The online version contains supplementary material available at 10.1186/s41016-022-00279-z.

## Background

Neurological neoplasms account for the majority of solid tumors in children and are the primary cause of malignancy-related deaths. In recent decades, advances in genetics and molecular biology have led to improvements in the diagnosis and treatment methods in pediatric neuro-oncology around the world. The latest WHO classification of tumors of the CNS has also improved the criteria for diagnosis [1]. China is a developing country with the largest population in the world, and there are many pediatric patients with neurological neoplasms. With the rapid increase and mobility in the population, we need to update the latest data. As the National Brain Tumor Registry of China is still under construction, we collected the latest data from Beijing Tiantan Hospital Pediatric Neurosurgery Department (China’s largest Pediatric Neurosurgery Center [2]), hoping to realize the population distribution characteristics of CNS tumors in children so as to lay a foundation for the follow-up clinical research.

## Methods

From January 2015 to December 2019, 3180 pediatric patients up to 15 years old underwent surgery to remove primary neurological neoplasms in Beijing Tiantan Hospital. The patients came from more than 30 provinces, municipalities, and autonomous regions in China. In this retrospective study, all patients were diagnosed histopathologically by the same group of pathologists from the Department of Pathology in Beijing Tiantan Hospital across the 5-year period. The histological diagnosis and tumor grade were assessed according to the 2016 WHO classification of CNS tumors [1]. WHO grade I and grade II indicate low-grade tumors, and WHO grades III and IV indicate high-grade tumors. Supratentorial tumors consist of tumors of the cerebral hemisphere, third ventricle and lateral ventricle, saddle region, and pineal region; infratentorial tumors include tumors of the cerebellum, brainstem, and fourth ventricle.

All patients were divided artificially into five age groups of 2-year intervals except the infant group (0-3 years of age): 4-6, 7-9, 10-12, and 13-15 years of age. For each patient with tumor recurrence whose pathological typing and/or grade changed, we remained the initial diagnosis to standardize the results. In terms of the latest classification, we adjusted some items in this study. For example, we included fibrillary astrocytoma as diffuse astrocytoma because their diagnoses are almost the same, and gliomatosis cerebri was deleted as a distinct entity [1, 3]. Due to the difficulty of popularizing molecular testing in developing countries, the diagnosis of all tumors was still based on morphologic features.

## Results

**Fig. 1:**
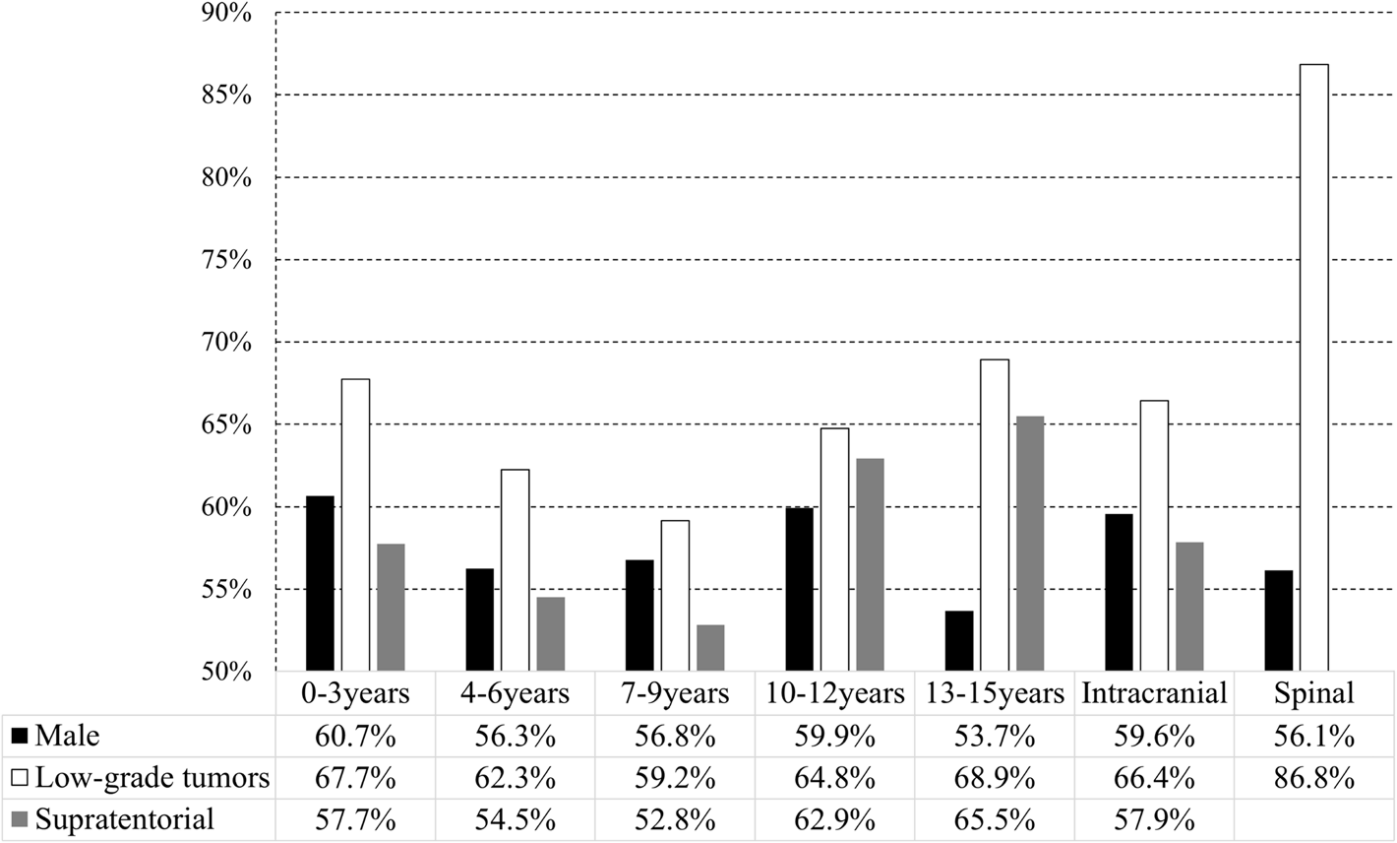
The percentage of male patients, supratentorial location, and low-grade tumors of neurological neoplasms

**Fig. 2:**
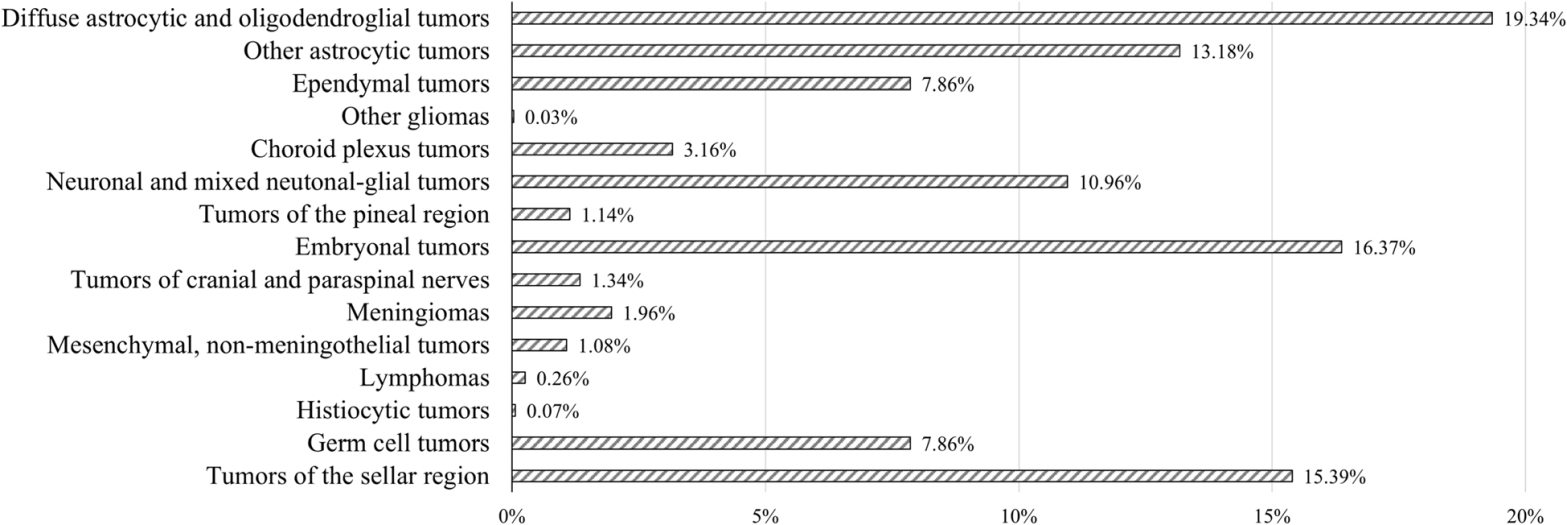
The percentage of various types of childhood intracranial tumors according to the 2016 WHO classification of central nervous system tumors

In 3180 children with CNS tumors, the prevalence of intracranial tumors was 96.4% (3066), while that of spinal tumors was 3.6% (114). The sex ratio of males to females was 1.47: 1. The majority of all patients had low-grade tumors, which accounted 66.4% (2036) in the brain and 86.8% (99) in the spinal cord. For intracranial tumor location, supratentorial (57.9%) tumors were slightly more preponderant than infratentorial tumors (42.1%). The percentages of affected male patients, low-grade CNS tumors, and supratentorial tumor location are shown for each age group, the intracranial tumor group, and the spinal tumor group (Fig. 1). Based on the 2016 WHO classification of CNS tumors, we listed the prevalence of each major group of tumors (Fig. 2). Diffuse astrocytic and oligodendroglial tumors (19.34%) accounted for the majority of tumors, followed by embryonal tumors (16.37%) and tumors of the sellar region (15.39%). Due to the large population of affected children, we observed diversity in the histopathology of neurological neoplasms and differences between intracranial and spinal tumors (Table 1).

**Table 1:** The histological types and frequency of intracranial (including supratentorial and infratentorial) and spinal tumors [*n*, *n* (%)]

Histological types	Intracranial		Spinal
	Supratentorial	Infratentorial	
Diffuse astrocytoma	118 (3.71)	184 (5.79)	6 (0.19)
Anaplastic astrocytoma	17 (0.53)	23 (0.72)	2 (0.06)
Glioblastoma	61 (1.92)	35 (1.10)	1 (0.03)
Diffuse midline glioma, H3K27M-mutant	4 (0.13)	30 (0.94)	3 (0.09)
Oligodendroglioma	9 (0.28)	2 (0.06)	-
Anaplastic oligodendroglioma	7 (0.22)	4 (0.13)	-
Oligoastrocytoma	37 (1.16)	29 (0.91)	1 (0.03)
Anaplastic oligoastrocytoma	17 (0.53)	16 (0.50)	-
Pilocytic astrocytoma	140 (4.40)	222 (6.98)	8 (0.25)
Subependymal giant cell astrocytoma	28 (0.88)	-	-
Pleomorphic xanthoastrocytoma	14 (0.44)	-	-
Subependymoma	1 (0.03)	2 (0.06)	-
Myxopapillary ependymoma	-	-	2 (0.06)
Ependymoma	34 (1.07)	56 (1.76)	9 (0.28)
Anaplastic ependymoma	57 (1.79)	91 (2.86)	2 (0.06)
Astroblastoma	1 (0.03)	-	-
Choroid plexus papilloma	53 (1.67)	11 (0.35)	-
Atypical choroid plexus papilloma	12 (0.38)	3 (0.09)	-
Choroid plexus carcinoma	17 (0.58)	1 (0.03)	-
Dysembryoplastic neuroepithelial tumor	86 (2.70)	3 (0.09)	-
Gangliocytoma	4 (0.13)	3 (0.09)	2 (0.06)
Ganglioglioma	81 (2.55)	19 (0.60)	5 (0.16)
Anaplastic ganglioglioma	-	1 (0.03)	1 (0.03)
Dysplastic cerebellar gangliocytoma (Lhermitte-Duclos disease)	-	2 (0.06)	-
Desmoplastic infantile astrocytoma and ganglioglioma	8 (0.25)	3 (0.09)	-
Papillary glioneuronal tumor	39 (1.23)	18 (0.57)	-
Central neurocytoma	6 (0.19)	-	-
Extraventricular neurocytoma	5 (0.16)	1 (0.03)	-
Paraganglioma	1 (0.03)	-	-
Mixed glioneuronal tumor	39 (1.23)	17 (0.53)	1 (0.03)
Pineocytoma	2 (0.06)	-	-
Pineal parenchymal tumor of intermediate differentiation	8 (0.25)	-	-
Pineoblastoma	22 (0.69)	-	-
Papillary tumor of the pineal region	3 (0.09)	-	-
Medulloblastoma	-	437 (13.74)	-
Embryonal tumor with multilayered rosettes	11 (0.35)	3 (0.09)	1 (0.03)
Medulloepithelioma	-	1 (0.03)	-
CNS neuroblastoma	2 (0.06)	1 (0.03)	-
CNS embryonal tumor, NOS	28 (0.88)	5 (0.16)	2 (0.06)
Atypical teratoid/rhabdoid tumor	8 (0.25)	5 (0.16)	1 (0.03)
CNS embryonal tumor with rhabdoid features	1 (0.03)	-	-
Neurofibroma	1 (0.03)	2 (0.06)	6 (0.19)
Perineurioma	8 (0.25)	30 (0.94)	20 (0.63)
Meningioma	59 (1.86)	1 (0.03)	6 (0.19)
Mesenchymal, non-meningothelial tumors	11 (0.35)	22 (0.69)	20 (0.63)
Lymphomas	6 (0.19)	2 (0.06)	1 (0.03)
Langerhans cell histiocytosis	2 (0.06)	-	-
Germinoma	35 (1.10)	2 (0.06)	-
Non-germinoma germ cell tumors	199 (6.26)	5 (0.16)	14 (0.44)
Craniopharyngioma	472 (14.84)	-	-

The five most frequent intracranial tumors were craniopharyngioma (15.4%), medulloblastoma (14.3%), pilocytic astrocytoma (11.8%), diffuse astrocytoma (9.8%), and anaplastic ependymoma (4.8%). These five tumors accounted for 56.1% of all intracranial tumors (1721/3066), and each tumor was more common in males than in females. The highest male-female ratio was observed in anaplastic ependymoma (2.08:1), followed by medulloblastoma (1.89:1). Regarding the tumor locations, the majority were supratentorial tumors, and the ratio of supratentorial tumors to infratentorial tumors was 1.37:1. However, in addition to medulloblastoma and craniopharyngioma (both located in the infratentorial or supratentorial region), pilocytic astrocytoma, diffuse astrocytoma, and anaplastic ependymoma were all more commonly found in the infratentorial region (Table 2). It is worth noting that there were only 37 cases of germinomas in this study, which does not indicate a sharp reduction in the incidence rate but rather a more effective and less harmful treatment strategy.

**Table 2:** Age-related frequency, sex ratio, histological, and topographical distribution of the 5 most common intracranial tumors [*n*, *n* (%)]. (*M* male, *F* female, *IT* infratentorial, *ST* supratentorial)

Histological types	Frequency	0–3 years	4–6 years	7–9 years	10–12 years	13–15 years	Ratios	
							M:F	IT:ST
Craniopharyngioma	472 (15.4)	70 (2.3)	149 (4.9)	109 (3.6)	88 (2.9)	56 (1.8)	1.37: 1	-
Medulloblastoma	437 (14.3)	57 (1.9)	136 (4.4)	137 (4.5)	73 (2.4)	34 (1.1)	1.89: 1	-
Pilocytic astrocytoma	362 (11.8)	77 (2.5)	110 (3.6)	75 (2.4)	58 (1.9)	42 (1.4)	1.14: 1	1.59: 1
Diffuse astrocytoma	302 (9.8)	72 (2.3)	81 (2.6)	59 (1.9)	54 (1.8)	36 (1.2)	1.32: 1	1.56: 1
Anaplastic ependymoma	148 (4.8)	45 (1.5)	54 (1.8)	29 (0.9)	12 (0.4)	8 (0.3)	2.08: 1	1.60: 1
Intracranial tumors	3066 (100)	556 (18.1)	818 (26.7)	687 (22.4)	553 (18.0)	452 (14.8)	1.47: 1	0.73: 1

Concerning the distribution of age across the common intracranial tumors, in all age groups, the prevalence of children diagnosed with medulloblastoma between 4-6 years old and 7-9 years old was almost the same. However, the other common intracranial tumors were highly prevalent among children in the 4-6 age group. Medulloblastoma was most common in male children (286 cases), while craniopharyngioma was most common in female children (199 cases). Unlike intracranial tumors, the most common spinal tumor was perineurioma (20 cases), followed by lipoma (13 cases), ependymoma (9 cases), pilocytic astrocytoma (8 cases), and mature teratoma (8 cases). The prevalence of spinal tumors increased with age. There were slightly more males with spinal tumors than females, and the ratio was 1.28: 1.

Although our study is based on a single center, 3180 pediatric patients came from more than 30 provincial administrative units. Shandong Province and Hebei Province had the most children with CNS tumors, with 470 (14.78%) and 460 (14.47%) cases, respectively, followed by Henan Province (10.24%), Anhui Province (6.29%), and Jiangsu Province (4.75%). The data showed that the time distributions of CNS tumors from various regions were different. The majority of pediatric patients in 2015, 2017, and 2019 came from Hebei Province, while most patients in 2016 and 2018 came from Shandong Province (Table 3). The rest data from other provinces are listed in Supplementary Table 1.

**Table 3:** Province and year distribution of childhood central nervous system tumors [*n*, *n* (%)]

Province	*n* (%)	2015y	2016y	2017y	2018y	2019y
Shandong	470 (14.78)	87 (2.73)	97 (3.05)	104 (3.27)	102 (3.21)	80 (2.52)
Hebei	460 (14.47)	88 (2.77)	82 (2.58)	108 (3.40)	85 (2.67)	97 (3.05)
Henan	326 (10.24)	79 (2.48)	70 (2.20)	68 (2.14)	58 (1.82)	51 (1.60)
Anhui	200 (6.29)	42 (1.32)	42 (1.32)	46 (1.45)	34 (1.07)	36 (1.13)
Jiangsu	151 (4.75)	32 (1.00)	37 (1.17)	24 (0.75)	27 (0.85)	31 (0.97)

## Discussion

From 2012 to 2016, the average annual incidence of primary CNS tumors for patients 19 years of age was 6.06 cases per 100,000 people in the USA [4]. Lower incidence rates were reported in other parts of the world, such as Japan (3.61 per 100,000 children) [5], Germany (2.6) [6], Italy (3.46) [7], and Taiwan (1.7) [8]. The reported incidence rate of CNS tumors increased slightly for those 0-19 years of age over the past 30 years in the USA [4, 9, 10]. This increase in the incidence is partly due to the improvements in diagnostic techniques, including magnetic resonance imaging (MRI), which has significantly enhanced the sensitivity of diagnosis [11–13]. Therefore, these findings illustrated the importance of the disease surveillance and registry system [11, 14] and reflected the demand for a registry system for CNS tumors among children (based on populations or hospitals) in China. Previous studies showed that pediatric intracranial tumors were more common in males [15–18]. We found a 1.47: 1 male-to-female ratio, indicating that male children were more likely to be affected, like previous literature reports. This predominance among male children was most evident in anaplastic ependymoma and medulloblastoma. However, there was a slightly higher incidence in female children in our study than in the study conducted in Beijing Tiantan Hospital from 2001 to 2005 [15].

Most of the pathological tumor types described in the 2016 WHO classification was observed in this retrospective study, demonstrating the diversity of CNS tumors among children. Studies have reported that the pathological types of intracranial tumors in children are different from those in adults. Meningiomas, pituitary tumors, and malignant gliomas are the most common types of adult intracranial tumors [19], whereas our study showed that craniopharyngioma was the most frequent type of intracranial tumor in children. Common intracranial tumors also included medulloblastoma, pilocytic astrocytoma, diffuse astrocytoma, and anaplastic ependymoma; the prevalence of these tumors herein was slightly different than those reported in other countries [4, 6, 7]. The most notable aspect of this study is the prevalence of GCTs, mostly observed in pediatric patients in Asia [20, 21]. However, during the period from 2015 to 2019, only 37 children were diagnosed with pure germinomas (1.16%) and treated with surgical resection. These data do not indicate a significant reduction in the prevalence of germinomas but rather improvements in the diagnosis and treatment of these tumors over the last two decades. Germinomas are extremely sensitive to radiotherapy, and a complete response could be achieved with radiotherapy alone in most cases [22, 23]; thus, pediatric patients with germinoma are more likely to avoid surgery except in emergency situations.

The overall data regarding age and tumor location showed that tumors were more commonly found in the supratentorial region than the infratentorial region in each age group. The largest difference was observed in the 13-15-year-old group. However, the most common intracranial tumors are found in the infratentorial region (except craniopharyngioma, which originates only in the sellar region). In the group aged 7-9 years old, infratentorial tumors accounted for the highest proportion, which may be due to the high incidence of medulloblastoma. An analysis of tumor grade and children’s age showed that most high-grade and low-grade tumors were found in children aged 4-6 years old, and the majority of tumors in each age group were low grade. This illustrated that the peak age of children with intracranial tumors is younger than in previous studies [15].

Regarding province and year distribution in childhood intracranial tumors, although the patients were from almost all the country’s provincial districts, they were not distributed evenly. Shandong Province and Hebei Province had the most pediatric patients, while Hong Kong and the Tibet Autonomous Region had the fewest patients. These results were related to the distance from Beijing, the level of medical care, and the economic level in the location of the patients. The overall trend over the 5-year period was not obvious. The number of patients was the largest in 2017, while there was a small decline in 2019. This trend may be related to the continuous development of medical criteria in various regions over recent years.

The research suggested that the CNS tumors are still one of the most important factors affecting children’s lives. Differences in age, region, and ethnicity may be potential factors affecting the pathological type and incidence rate of intracranial tumors in children [4, 10]. Among the top five most common types of intracranial tumors in this research, the majority were low-grade tumors, and the ratio of low-grade tumors to high-grade tumors was 1.94: 1 (1136/585). Among these intracranial tumors, craniopharyngioma, pilocytic astrocytoma, and diffuse astrocytoma are all low-grade tumors. This finding was related to the polymorphism of children’s tumor genes and the location of tumor origins [24]. Due to the popularization of neuroimaging technologies [13, 24], children with intracranial brain tumors can be diagnosed and treated earlier and more precisely; however, the prognosis and quality of life of children still need to be further analyzed.

Based on these findings, we are looking forward to multicenter and multidisciplinary collaborative research. Combining domestic regional characteristics to establish the disease surveillance and registry system and to improve epidemiological follow-up practices will be significant for the diagnosis and treatment of intracranial tumors among children in China.

## Conclusions

First, the complete follow-up and registry system in China is still under construction, so not all cases in our study have follow-up results. And second, molecular genotyping has gradually gained popularity recently, but it has not been analyzed in the early cases because of the restriction of cost and time. Thus, we could not completely keep to the latest WHO classification. Beijing Tiantan Hospital has the largest pediatric neurosurgery center in China [2], although it cannot represent the national data, we believe that it is still of necessary research significance, to establish a national follow-up and registry system in the future. In summary, more in-depth researches by providing an overview of epidemiological information on primary brain tumors were needed to promote multidisciplinary research [24].

## Supplementary Information

**Additional file 1.** Province and year distribution of childhood central nervous system tumors [n, n (%)].

## Data Availability

The datasets generated during and/or analyzed during the current study are available from the corresponding author on reasonable request.
