# Supplementary material for: Epidemiological characteristics of central nervous system tumors in children: a 5-year review of 3180 cases from Beijing Tiantan Hospital
Source: Chin Neurosurg J. 2022 May 12;8:11. doi: 10.1186/s41016-022-00279-z (PMC9096059; doi:10.1186/s41016-022-00279-z)
Supplement: Supplementary file 1 — Additional file 1. Province and year distribution of childhood central nervous system tumors [n, n (%)]. [file 41016_2022_279_MOESM1_ESM.docx]

**Table 1.** Province and year distribution of childhood central nervous system tumors [n, n (%)].

| Province | n (%) | 2015y | 2016y | 2017y | 2018y | 2019y |
| --- | --- | --- | --- | --- | --- | --- |
| Shandong | 470 (14.78) | 87 (2.73) | 97 (3.05) | 104 (3.27) | 102 (3.21) | 80 (2.52) |
| Hebei | 460 (14.47) | 88 (2.77) | 82 (2.58) | 108 (3.40) | 85 (2.67) | 97 (3.05) |
| Henan | 326 (10.24) | 79 (2.48) | 70 (2.20) | 68 (2.14) | 58 (1.82) | 51 (1.60) |
| Anhui | 200 (6.29) | 42 (1.32) | 42 (1.32) | 46 (1.45) | 34 (1.07) | 36 (1.13) |
| Jiangsu | 151 (4.75) | 32 (1.00) | 37 (1.17) | 24 (0.75) | 27 (0.85) | 31 (0.97) |
| Shanxi | 148 (4.65) | 34 (1.07) | 31 (0.97) | 36 (1.13) | 26 (0.82) | 21 (0.66) |
| Beijing | 141 (4.43) | 20 (0.63) | 21 (0.66) | 24 (0.75) | 15 (0.47) | 61 (1.92) |
| Inner Mongolia Municipality | 124 (3.90) | 27 (0.85) | 30 (0.94) | 27 (0.85) | 18 (0.57) | 22 (0.69) |
| Liaoning | 105 (3.30) | 23 (0.72) | 17 (0.53) | 29 (0.91) | 20 (0.63) | 16 (0.50) |
| Heilongjiang | 100 (3.14) | 21 (0.66) | 20 (0.63) | 26 (0.82) | 17 (0.53) | 16 (0.50) |
| Jiangxi | 83 (2.62) | 18 (0.57) | 13 (0.41) | 18 (0.57) | 20 (0.63) | 14 (0.44) |
| Zhejiang | 82 (2.58) | 22 (0.69) | 14 (0.44) | 16 (0.50) | 14 (0.44) | 16 (0.50) |
| Hubei | 75 (2.36) | 10 (0.31) | 17 (0.53) | 23 (0.72) | 12 (0.38) | 13 (0.41) |
| Gansu | 69 (2.17) | 12 (0.38) | 12 (0.38) | 20 (0.63) | 15 (0.47) | 10 0.31) |
| Fujian | 68 (2.14) | 14 (0.44) | 14 (0.44) | 15 (0.47) | 14 (0.44) | 11 (0.35) |
| Hunan | 65 (2.04) | 20 (0.63) | 14 (0.44) | 9 (0.28) | 12 (0.38) | 10 0.31) |
| Jilin | 64 (2.01) | 12 (0.38) | 19 (0.60) | 14 (0.44) | 11 (0.35) | 8 (0.25) |
| Guangdong | 62 (1.95) | 7 (0.22) | 10 0.31) | 15 (0.47) | 17 (0.53) | 13 (0.41) |
| Chongqing | 54 (1.70) | 7 (0.22) | 16 (0.50) | 12 (0.38) | 9 (0.28) | 10 (0.31) |
| Shaanxi | 53 (1.67) | 15 (0.47) | 10 0.31) | 15 (0.47) | 5 (0.13) | 8 (0.25) |
| Guizhou | 52 (1.64) | 7 (0.22) | 9 (0.28) | 14 (0.44) | 14 (0.44) | 8 (0.25) |
| Sichuan | 49 (1.54) | 12 (0.38) | 8 (0.25) | 12 (0.38) | 11 (0.35) | 6 (0.19) |
| Yunnan | 40 (1.26) | 2 (0.06) | 8 (0.25) | 7 (0.22) | 12 (0.38) | 11 (0.35) |
| Guangxi Zhuang Autonomous Region | 33 (1.04) | 5 (0.16) | 12 (0.38) | 6 (0.19) | 4 (0.09) | 6 (0.19) |
| Tianjin | 32 (1.00) | 7 (0.22) | 5 (0.16) | 8 (0.25) | 5 (0.16) | 7 (0.22) |
| Qinghai | 19 (0.60) | 4 (0.13) | 5 (0.16) | 2 (0.06) | 4 (0.13) | 4 (0.13) |
| Xinjiang Uygur Autonomous Region | 18 (0.57) | 0 | 1 (0.03) | 5 (0.16) | 5 (0.16) | 7 (0.22) |
| Ningxia Hui Autonomous Region | 15 (0.47) | 1 (0.03) | 7 (0.22) | 3 (0.09) | 2 (0.06) | 2 (0.06) |
| Shanghai | 11 (0.35) | 2 (0.06) | 3 (0.09) | 3 (0.09) | 0 | 3 (0.09) |
| Hainan | 8 (0.25) | 1 (0.03) | 4 (0.13) | 1 (0.03) | 1 (0.03) | 1 (0.03) |
| Tibet Autonomous Region | 2 (0.06) | 1 (0.03) | 0 | 0 | 1 (0.03) | 0 |
| Hong Kong | 1 (0.03) | 0 | 0 | 1 (0.03) | 0 | 0 |
